# Induction of Endoplasmic Reticulum Stress by CdhM Mediates Apoptosis of Macrophage During *Mycobacterium tuberculosis* Infection

**DOI:** 10.3389/fcimb.2022.877265

**Published:** 2022-04-04

**Authors:** Peng Xu, Jing Tang, Zheng-Guo He

**Affiliations:** ^1^ College of Life Science and Technology, Huazhong Agricultural University, Wuhan, China; ^2^ State Key Laboratory for Conservation and Utilization of Subtropical Agro-Bioresources, College of Life Science and Technology, Guangxi University, Nanning, China

**Keywords:** Mycobacterium, CdhM, host–pathogen interaction, ER stress, UPR, apoptosis

## Abstract

The normal operation of the endoplasmic reticulum (ER) is critical for cells and organisms. However, ER stress, caused by imbalanced protein folding, occurs frequently, which perturbs the function of the ER and even results in cell apoptosis eventually. Many insults can induce ER stress; pathogen infection is one of them. Most of the genes involved in ER stress have been reported to be upregulated in *Mycobacterium tuberculosis* (Mtb) granulomas of humans and mice, implicating that infection with Mtb can induce ER stress. However, little is known about the molecular mechanism of Mtb induction of ER stress. Here, we reveal that *Mycobacterium* protein CDP-diglyceride hydrolase of Mycobacteriumn (CdhM) could target the ER and cause abnormal ER morphology and cell death. RNA-seq analysis suggests that most of the ER stress-involved genes were modulated by CdhM. Further assessed by biochemical experiments, the transcription and protein levels of ER stress markers BiP and CHOP, as well as the levels of XBP1 splicing and eIF2α phosphorylation, were significantly increased by CdhM, confirming that CdhM could induce ER stress alone or during infection. A single conserved amino acid mutant of CdhM, including L44A, G96A, H150A, and W175A, was incapable of inducing ER stress, which indicates that induction of ER stress by CdhM is specific and functional. Furthermore, CdhM-induced ER stress could also promote apoptosis of macrophages during Mtb infection. Overexpression of CdhM conferred a significant benefit for Mtb replication by releasing Mtb into extracellular during infection of macrophage *in vitro*, as presented in CFU assays. Overall, our study identified a novel Mtb effector protein CdhM which may promote Mtb dissemination and proliferation by induction of ER stress and apoptosis and provided new insight into the physiological significance of induction of ER stress in tuberculosis (TB) granulomas.

## Introduction

The endoplasmic reticulum (ER) is a complex and large structure in the cytoplasm and spans between the nucleus and the cell membrane. It plays important roles in protein synthesis, protein folding, and posttranslational modification, as well as lipid synthesis and calcium storage (Marciniak and Ron, 2006; Puzianowska-Kuznicka and Kuznicki, 2009; Balla et al., 2020). It is therefore crucial for cells and even for organisms that the ER works normally. However, when unfolded or misfolded proteins accumulate in the ER, the cell experiences ER stress (Ron and Walter, 2007), which will lead to dysfunction of the ER. If the ER stress cannot be restored and is beyond the tolerance of cells, programmed cell death will happen (Tabas and Ron, 2011; Walter and Ron, 2011). Multiple insults disrupt the process of protein folding in the ER and cause ER stress, such as toxins, energy deprivation, alterations in redox state, and changes in calcium concentration (Celli and Tsolis, 2015; Marciniak et al., 2021).

Once ER stress occurs, an evolutionarily conserved cytoprotective signaling pathway called the unfolded protein response (UPR) will be activated to relieve physiological stress on the ER and maintain cellular homeostasis (Walter and Ron, 2011; Gardner et al., 2013). The UPR is composed of at least three mechanistically distinct branches which are initiated by three different sensors, namely, inositol-requiring enzyme 1 (IRE1), protein kinase RNA-like endoplasmic reticulum kinase (PERK), and activating transcription factor 6 (ATF6) (Mori et al., 1993; Yoshida et al., 1998; Harding et al., 1999). The three sensors are bound by the ER chaperone immunoglobulin-binding protein (BiP) to keep silent under normal state but are activated by ER stress coupled with dissociation with BiP (Shiu et al., 1977; Hetz, 2012). Activated IRE1 activates the transcription factor XBP1 *via* an unconventional splicing and reduces the synthesis of protein by degrading ER-localized mRNA transcripts (Mori et al., 1993; Lin et al., 2007; Uemura et al., 2009). Active PERK phosphorylates eIF2α to inhibit the translation of protein directly. On the other hand, P-eIF2α can also promote the translation of the transcription factor ATF4 (Harding et al., 1999; Hetz, 2012). Activated ATF6 is trafficked to the Golgi where it is proteolytically processed to release a nuclear targeted transcription factor domain (Yoshida et al., 1998). XBP1, ATF4, and cleaved ATF6 regulate the expression of numerous UPR target genes to respond to ER stress (Hetz, 2012).

Pathogen infection causing ER stress has been widely reported in bacteria, viruses, fungi, and protozoan parasites (Celli and Tsolis, 2015; Li et al., 2015; Galluzzi et al., 2017), such as *Brucella* spp. (de Jong et al., 2013; Roy et al., 2013), *Legionella* spp. (Hempstead and Isberg, 2015), *Escherichia* spp. (Paton et al., 2006), *Helicobacter* spp. (Zhu et al., 2017), *Listeria* spp. (Pillich et al., 2012), *Chlamydia* spp. (Webster et al., 2016), *Aspergillus fumigatus* (Lee et al., 2016), and a number of viruses (Jheng et al., 2014; Li et al., 2015). As a matter of course, *Mycobacterium*, a well-known intracellular pathogen, can also induce ER stress during infection. It was reported for the first time in 2010 that most of the genes involved in ER stress were upregulated in *Mycobacterium tuberculosis* (Mtb) granulomas of humans and mice (Seimon et al., 2010). Subsequently, *Mycobacterium bovis* (Cui et al., 2016), *Mycobacterium smegmatis* (Kim et al., 2018), and *Mycobacterium avium* (Go et al., 2019) were identified to elicit ER stress during infection as well. Moreover, the molecular mechanism of Mtb proteins HBHA and Rv0297 inducing ER stress has been characterized (Choi et al., 2013; Grover et al., 2018). However, it is still necessary to investigate the thorough relationship of Mtb and ER stress through which the therapeutic schedules of sophisticated tuberculosis may benefit.

In the present study, we identified another Mtb effector protein, termed CdhM, which can localize to the ER and cause abnormal ER morphology and cell death. Subsequent RNA-seq analysis and biochemical assays proved that CdhM elicits ER stress alone or during infection. Additionally, CdhM can also promote the ER stress-mediated apoptosis of macrophages during Mtb infection, which is considered to facilitate the dissemination of Mtb during infection.

## Materials and Methods

### Culture Conditions of Bacterial Strains and Mammalian Cells

*Escherichia coli* DH5α used for gene cloning was cultured in Luria-Bertani medium with or without agar at 37°C. *Mycobacterium tuberculosis* H37Ra was used as a model strain in this study and was grown in Middlebrook 7H9 medium (BD Biosciences, Franklin Lakes, NJ, USA) supplemented with 10% (v/v) OADC (oleic acid–albumin–dextrose–catalase; BD Biosciences), 0.2% (v/v) glycerol, and 0.05% (v/v) Tween-80 at 37°C. HEK293T, HeLa, and RAW264.7 obtained from the American Type Culture Collection (ATCC) were grown in DMEM (Gibco, Grand Island, NY, USA) medium supplemented with 10% fetal bovine serum (Gibco), 100 U/ml penicillin, and 100 μg/ml streptomycin (Gibco) at 37°C with 5% CO_2_.

### Plasmids and Recombinant Strain Construction

The plasmids below were purchased from Clontech (Mountain View, CA, USA): pEGFP-C1, pDsRed2-ER, pEGFP-Actin, and pEYFP-Mem. CdhM and its mutants were cloned into pEGFP-C1 at restriction sites of HindIII and PstI and selected by kanamycin. The pMind vector was used for CdhM knockout in H37Ra as described previously (Liu et al., 2019). pMV261 was used for gene overexpression in H37Ra (Liu et al., 2019). All constructs were examined by sequencing. Plasmids were electroporated into H37Ra and selected on 7H10 medium containing 50 µg/ml kanamycin.

### Imaging of Live Cells

HEK293T or HeLa cells were seeded into 96-well plates a day before transfection, and transfection was performed when confluence reached around 90%. Hieff Trans™ Liposomal Transfection Reagent (YEASEN Biotech, Shanghai, China) was used for transfection. The plasmids without endotoxin were prepared using the EndoFree Maxi Plasmid Kit (TIANGEN Biotech, Beijing, China). The experimental procedures followed the respective manufacturer’s instructions. Around 24 h post transfection, cells were subcultured into the tailor-made 96-well plates with serial dilutions for live-cell imaging using the High Content Imaging System (PerkinElmer, Waltham, MA, USA). The High Content Imaging System could provide suitable conditions, like 37°C with 5% CO_2_, for normal cell growth, and trace multiple precise fields of view continuously.

### RNA-seq

HEK293T cells were seeded into 12-well plates a day before transfection and transfection performed when confluence reached around 90% as above. 48 hours post transfection, cells were washed for a time with PBS and lysed directly with TRIzol reagent (Invitrogen, Carlsbad, CA, USA) followed by flash freezing with liquid nitrogen. Then, samples were sent to Novogene (Novogene, Beijing, China) with dry ice for RNA isolation and sequencing as well as bioinformatics analysis. For RAW264.7, cells were seeded into 12-well plates a day before infection. Then, RAW264.7 cells were infected with CdhM-related strains at a multiplicity of infection (MOI) of 10 and incubated at 37°C with 5% CO_2_ for 24 h. Subsequent procedures were the same as HEK293T.

### RNA Isolation and Quantitative Real-Time PCR

The protocols for transfection or infection were the same as the sample preparation of RNA-seq. Total RNA was isolated from the transfected or infected cells using TRIzol reagen (Invitrogen). The quality of RNA was assessed using NanoDrop 2000 (Thermo Fisher, Waltham, MA, USA). MonScript™ RTIII Super Mix with dsDNase (Monad Biotech, Shanghai, China) was used for reverse transcription according to the manufacturer’s protocol. Then, quantitative real-time PCR (qRT-PCR) was performed for quantification of target gene expression with the SYBR Green PCR mixture (Aidlab, Beijing, China) in Bio-Rad CFX Connect Real-Time System. The relative mRNA expression levels were calculated by normalization to GAPDH. The primers used in this study are listed in **Supplementary Table 2**.

### Western Blot

Cells were seeded into 6-well plates a day before infection or transfection. The protocols for infection or transfection were the same as above. Then cells were washed three times with cold PBS and harvested from plates to microtubes followed by centrifugation at 1,000 rpm for 5 min. Cell samples were lysed with RIPA lysis buffer (Boster, Pleasanton, CA, USA) supplemented with protease inhibitor cocktail (GlpBio, Montclair, CA, USA) for 1 h on ice followed by centrifugation at 14,000 rpm for 15 min. Then, the supernatant was transferred into new microtubes and the concentration of total proteins was determined by Pierce™ BCA Protein Assay Kit (Thermo Fisher). Proteins were electrophoresed in 12% SDS-PAGE and transferred to a nitrocellulose blotting membrane (GE Healthcare Life Sciences, Chicago, IL, USA). The NC membrane was blocked with 5% BSA in TBST (20 mM Tris–HCl, pH 7.5, 137 mM NaCl, and 0.05% Tween 20) for 2 h at room temperature. Then the blots were incubated with primary rabbit or mouse IgG antibodies, with 1,000 times dilution in 5% BSA, overnight at 4°C, and then incubated with HRP-conjugated goat anti-rabbit or anti-mouse IgG secondary antibodies with 5,000 times dilution in 5% BSA for 2 h. The immunoblots were visualized with Immobilon Western Chemiluminescent HRP Substrate (Millipore, Burlington, MA, USA) according to the manufacturer’s protocol. ImageJ software was used for quantification of immunoblots. β-Actin was used as loading controls. All antibodies are listed as follows: BiP (CST#3177), CHOP (CST#2895), P-eIF2α (CST#3398), and β-actin (CST#8457) were purchased from Cell Signaling Technology (Danvers, MA, USA), and HRP-conjugated goat anti-rabbit (BA1054) and HRP-conjugated goat anti-mouse (BA1050) were purchased from Boster Biological Technology Co., Ltd. (Wuhan, China).

### Analysis of Cell Apoptosis

Cells were seeded into 12-well plates a day before infection. The protocols for infection were the same as above. Then cell apoptosis was detected with FITC Annexin V Apoptosis Detection Kit (BD, USA) according to the manufacturer’s protocol, and analysis was performed by flow cytometry (CytoFLEX LX, Beckman, Brea, CA, USA). The principles of this kit are that externalized membrane phospholipid phosphatidylserine (PS), which is a hallmark of apoptotic cells, will be stained with FITC-labeled annexin V protein, and propidium iodide (PI) can only be used to stain the nuclei of dead or membrane damaged cells which occur at the late stage of apoptosis. Thus, early apoptosis cells (FITC Annexin V positive and PI negative) and late apoptosis cells (FITC Annexin V and PI positive) can be distinguished from viable (FITC Annexin V and PI negative) or mechanically damaged cells (FITC Annexin V negative and PI positive).

### CFU Determination of Mtb During Infection of Macrophage

RAW264.7 cells were seeded into 24-well plates a day before infection. Then it was infected with CdhM-related strains at an MOI of 10 and incubated for 4 h at 37°C with 5% CO_2_. After 4 h for phagocytosis, the cells were washed three times with PBS to remove extracellular bacteria, and then a fresh medium with or without gentamycin was added for subsequent cell culture. At 4, 24, and 48 h postinfection (hpi), all bacteria, including intracellular and extracellular, were collected and plated on 7H10 (BD Biosciences) agar plates with serial dilutions. After being incubated at 37°C for about 3 weeks, colonies were counted.

### Growth Curve and Colony Morphology of Mtb

CdhM-related strains were grown in Middlebrook 7H9 medium (BD, USA) supplemented with 10% (v/v) OADC (oleic acid–albumin–dextrose–catalase; BD Biosciences), 0.2% (v/v) glycerol, and 0.05% (v/v) Tween 80, at 37°C and 180 rpm. The OD_600_ of bacteria was measured with a day interval. The mid-logarithmic phase of bacteria was seeded on 7H10 (BD, USA) agar plates for observation of colony morphology.

### Statistical Analysis

Statistical analysis was performed using GraphPad Prism 8.0 by one-way or two-way ANOVA followed by Tukey’s multiple comparisons test. Significant differences are marked with ∗p < 0.05, ∗∗p < 0.01, and ∗∗∗p < 0.001. All results are graphed as means ± SD for triplicate samples.

## Results

### CdhM Targets the ER and Causes Abnormal ER Morphology and Cell Death

**Figure 1:**
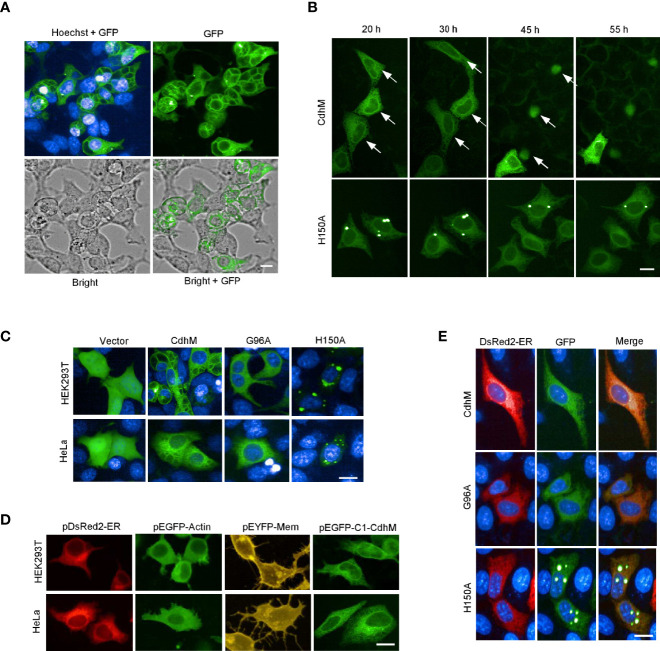
Phenotypes and subcellular localization of CdhM in host cells. **(A)** Cell morphology of HEK293T and subcellular localization of CdhM. HEK293T was transfected with CdhM labeled with GFP. **(B)**. Time-lapse live imaging to trace the behavior of the cells transfected with GFP-CdhM. See also **Supplementary Movies 1**–**4**. **(C)** Subcellular localization of CdhM mutants. All mutants were labeled with GFP similar to wild-type CdhM using vector pEGFP-C1. Plasmid was transfected into HEK293T and HeLa by liposome. The results of L44A and W175A were similar to G96A, so only the representative results of G96A were presented (the same below). **(D)** Comparison of subcellular localization of CdhM with several known subcellular makers. pDsRed2-ER, pEGFP-Actin, and pEYFP-Mem are commercially available vectors that can be used for fluorescent labeling of ER, actin, and cell membrane, respectively. **(E)** Co-localization of CdhM and the ER. pDsRed2-ER and pEGFP-C1-CdhM/G96A/H150A were co-transfected into HeLa. Confocal microscopy was used. Imaging of live cells was performed using the High-Content Imaging System (PerkinElmer). Hoechst (blue) was used for nuclear staining. Scale bar, 10 µm.

A high-throughput screening about the interaction of Mtb secretion proteins and macrophage had been performed in our lab (data not shown). A protein, which is annotated as CDP-diacylglycerol pyrophosphatase (alternative named CDP-diglyceride hydrolase) in KEGG, hereafter designated CdhM, caught our attention. We wondered therefore what will happen if CdhM is transfected into host cells. Surprisingly, a phenotype of intracellular vacuolation surrounding nuclei was observed at about 20 h post transfection (**Figure 1A**). Then, time-lapse live imaging was applied to trace the cells transfected with GFP-CdhM from 20 to 55 h post transfection, and we found that transfection with CdhM caused cell death with condensation morphology eventually, which resembled apoptosis (**Figure 1B**). We asked if those phenotypes are specific and functional. Therefore, the conserved amino acids of CdhM among all homologous sequences were analyzed by ClustalW Multiple Alignment and ten amino acids were identified (**Supplementary Figure 1**). A single conserved amino acid mutant of CdhM was constructed and transfected into host cells. The mutants, including L44A, G96A, H150A, and W175A, were found unable to cause vacuolation and condensation-like cell death anymore. Among these mutants, L44A, G96A, and W175A still kept the similar subcellular localization with wild-type CdhM, but H150A made an obvious difference (The results of L44A and W175A were similar to G96A, so only the representative results of G96A were presented, similarly in the following.) (**Figures 1B, C** and **Supplementary Movies 1**–**4**). It suggests that the phenotypes elicited by CdhM are specific and functional. Then, we compared the subcellular localization of CdhM with several known subcellular markers, such as pEGFP-Actin, pYFP-Membrane, and pDsRed2-ER, which are commercially available, and found that GFP-CdhM has a similar subcellular localization with DsRed2-ER (**Figure 1D**). The subsequent co-localization experiment verified that CdhM and its mutants L44A, G96A, and W175A, except H150A, localized to the ER (**Figure 1E**). We presumed therefore that the phenotype of intracellular vacuolation might be abnormal morphology of the ER, and the result of the condensation-like cell death might be apoptosis mediated by ER stress (Tabas and Ron, 2011).

### RNA-Seq of HEK293T Characterizes the Signaling Pathway Affected by CdhM

**Figure 2:**
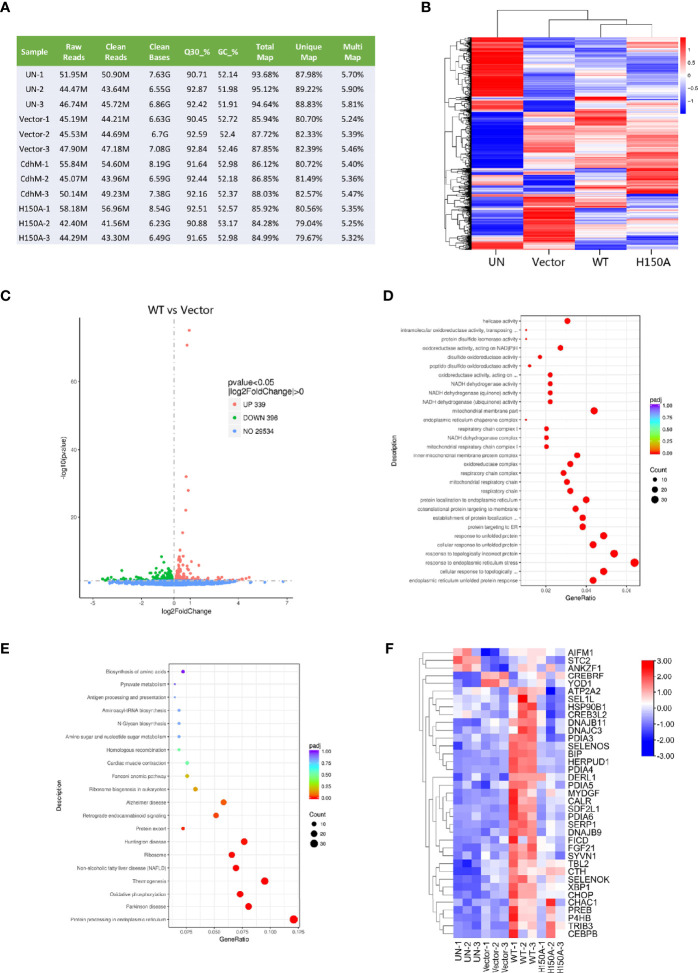
RNA-Seq of HEK293T transfected with CdhM. **(A)** Quality control and statistics for the RNA-Seq data of HEK293T. Each group with three parallel replicates. M, million. Q30%, the percentage of nucleotides with a quality value of 30. GC %, the percentage of guanine and cytosine in the cleaned reads. **(B)** Clustering heatmap of differential gene expressions. **(C)** Volcano plot shows differential gene expression between CdhM and empty vector. Gene Ontology (GO) **(D)** and KEGG **(E)** enrichment analysis of differentially expressed genes between CdhM and empty vector were implemented by the clusterProfiler R package, in which gene length bias was corrected. **(F)** Clustering heatmap analysis of DEGs involved in ER stress (p < 0.05). UN, un-transfected. Vector, empty pEGFP-C1 vector. WT, pEGFP-C1-CdhM. H150A, pEGFP-C1-H150A.

In order to uncover the potential molecular mechanism of the phenotypes caused by CdhM, a global transcriptome sequencing (RNA-seq) of HEK293T transfected with CdhM was performed. The cells of untransfected (UN), transfected with empty vector (vector), and mutant H150A were set as controls. The quality control and statistics of the RNA-Seq results suggest that the data are reliable (**Figure 2A**). A clustering heatmap of differential gene expression among all four groups indicated that significant differences in gene expression profiling do exist (**Figure 2B**). The differentially expressed genes (DEGs) between CdhM and empty vector were analyzed and shown on a volcano plot (**Figure 2C**). There are 339 genes upregulated and 396 genes downregulated significantly in the cells transfected with CdhM relative to the empty vector. Then Gene Ontology (GO) and KEGG enrichment analyses of these DEGs were implemented, and we found that the top biological processes or signaling pathways are mostly ER-related (**Figures 2D, E**). Hence, we speculated that transfection with CdhM might disrupt the normal function of the ER and that the cells were experiencing ER stress. A clustering heatmap analysis of the 37 DEGs involved in ER stress showed significant differences between CdhM and other control groups (**Figure 2F**). It is consistent with the results in Mtb granulomas (Seimon et al., 2010). Incorporation of the subcellular localization, morphology phenotyping, and transcriptome analysis suggest that CdhM may induce ER stress in host cells.

### Biochemical Evidence for CdhM Inducing ER Stress

**Figure 3:**
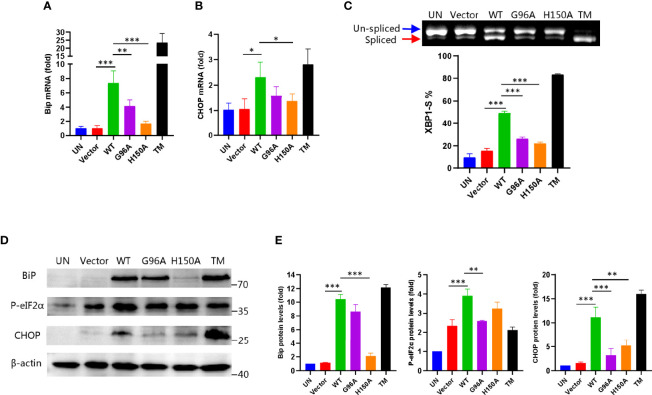
Biochemical evidence for CdhM induction of ER stress. Transcript levels of ER stress marker BiP **(A)** and CHOP **(B)** were measured by qRT-PCR. GAPDH was used as internal reference. **(C)** XBP1 splicing was analyzed by RT-PCR. Percent of XBP1-S (spliced XBP1) in total XBP1 was calculated and presented on the bottom. **(D)** Protein levels of ER stress marker BiP, CHOP, and phosphorylated eIF2α were determined by Western blot. β-Actin was used as internal reference. **(E)** Relative quantification of protein levels in **(D)**. UN, untransfected. Vector, empty pEGFP-C1 Vector. WT, pEGFP-C1-CdhM. G96A, pEGFP-C1-G96A. H150A, pEGFP-C1-H150A. TM, tunicamycin (positive control). *p < 0.05, **p < 0.01, ***p < 0.001 by one-way ANOVA. Means ± SD were shown (n = 3). Data are representative from at least three repetitions.

Based on the above results, we performed more biochemical experiments to validate if CdhM causes ER stress in host cells. As mentioned before, the UPR would be activated when the ER stress happened. BiP, in which the expression was upregulated and dissociated with three sensors of the UPR, is the marker of UPR initiation (Hetz, 2012). On the other hand, activation of the C/EBP homology protein (CHOP) is a marker of the late stage of the UPR, which implicates that cell apoptosis would happen (Tabas and Ron, 2011). Both proteins are therefore well-known indicators for assessing ER stress. Thus, we detected both transcriptional and protein levels of BiP and CHOP *via* qRT-PCR and Western blot, respectively. As shown in **Figures 3A, B, D, E**, compared with the empty vector, cells were transfected with CdhM, but its mutants, both BiP and CHOP, were upregulated significantly at both transcriptional and protein levels. It is consistent with the results of RNA-seq. After that, we wondered which signaling pathway of the three of the UPR was activated by CdhM. An unconventional splicing of XBP1 mRNA is the marker for the activation of the IRE1 pathway. A 26-base-pair fragment of XBP1 mRNA would be deleted by the activated IRE1 during the splicing process (Uemura et al., 2009). Thus, the PCR products based on the templates of spliced or un-spliced XBP1 cDNA could be separated by SDS-PAGE, and the percentage of spliced XBP1 in total XBP1 could be used to determine the degree of IRE1 activation. Experimental results show that the percentage of spliced XBP1 in the cells transfected with CdhM is about 50%, which is markedly higher than the empty vector as well as its mutants (**Figure 3C**), indicating that the IRE1-pathway was activated by CdhM. In addition, we also assessed the activation of the PERK pathway *via* the phosphorylation of eIF2α which is considered as the hallmark of PERK activation (Harding et al., 1999). Indeed, compared with the empty vector and its mutants, transfection with CdhM elevated the phosphorylation levels of eIF2α remarkably (**Figures 3D, E**). It implicated that the PERK pathway was activated by CdhM as well. Taken together, we confirmed that CdhM causes ER stress and activates the UPR through at least two signaling pathways.

### CdhM Promotes ER Stress During Mtb Infection of Macrophage

**Figure 4:**
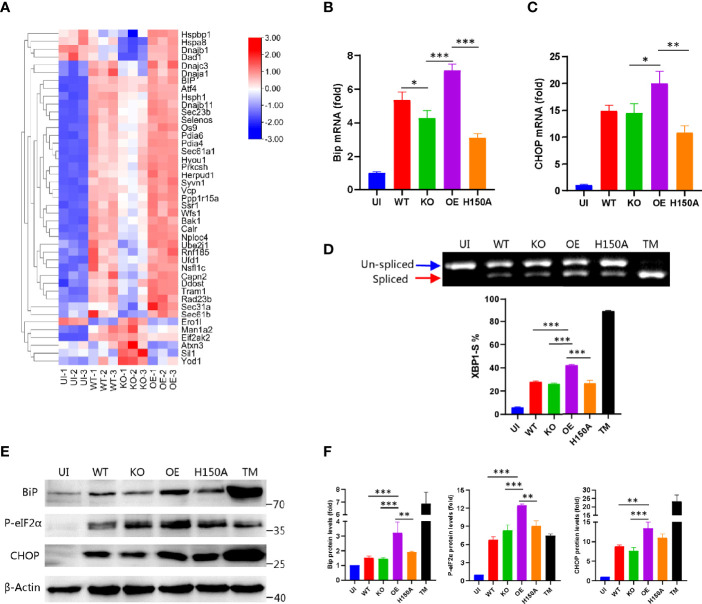
CdhM promotes ER stress during infection. **(A)** RNA-seq heatmap analysis of the genes involved in ER stress when RAW264.7 was infected by CdhM-related strains. Transcript levels of ER stress marker BiP **(B)** and CHOP **(C)** were assessed by qRT-PCR. GAPDH was used as internal reference. **(D)** XBP1 splicing was analyzed by RT-PCR. Percent of XBP1-S (spliced XBP1) in total XBP1 was calculated and presented on the bottom. **(E)** Protein levels of ER stress markers BiP, CHOP, and phosphorylated eIF2α were detected by Western blot. β-Actin was used as internal reference. **(F)** Relative quantification of protein levels in **(E)**. UI, uninfected. WT, wild-type H37Ra. KO, CdhM knockout strain. OE, CdhM overexpression strain. H150A, CdhM knockout strain complement with H150A mutant. TM, tunicamycin (positive control). *p < 0.05, **p < 0.01, ***p < 0.001 by one-way ANOVA. Means ± SD were shown (n = 3). Data are representative from at least three repetitions.

Having shown the ability of CdhM to target the ER and induce ER stress alone, we then considered if CdhM would exhibit these abilities during Mtb infection of macrophage. Hence, CdhM-related strains were prepared, such as CdhM-knockout strain (KO), constructed by double exchanges of homologous strands, CdhM-overexpression strain (OE), and mutant of H150A (**Supplementary Figure 2A, B**). Firstly, a transcriptome sequencing of RAW264.7 infected with wild-type (WT) and CdhM-related strains was performed (**Supplementary Table 1**). A clustering heatmap analysis of 43 DEGs involved in ER stress showed significant differences between KO and WT as well as OE, but the fold changes of the DEGs were not as large as these between the uninfected group (UI) and WT (**Figure 4A**). It suggests that besides CdhM there are other effectors of Mtb contributing to induction of ER stress during infection, which corresponds to the previous research (Choi et al., 2013; Grover et al., 2018). Next, biochemical experiments were performed to investigate the effect of CdhM in ER stress induction during Mtb infection of macrophage. The same as above, we measured the transcriptional and protein levels of ER stress markers BiP and CHOP (**Figures 4B, C, E, F**), and splicing of XBP1 (**Figure 4D**) as well as phosphorylation of eIF2α (**Figures 4E, F**). As shown in the results, compared with wild type and uninfected, overexpression of CdhM, except its mutants, significantly increased the expression levels of BiP and CHOP as well as phosphorylation levels of eIF2α and percentages of spliced XBP1. These results confirmed that CdhM worked in induction of ER stress during Mtb infection of macrophage. However, we were surprised that the CdhM-knockout strain showed no significant differences with the wild type. There are no redundant homologous genes of CdhM in the genome of Mtb. It seems that there were other effectors that compensated for the lack of CdhM in induction of ER stress during Mtb infection of host cells. Collectively, CdhM can also induce ER stress during Mtb infection of the host.

### CdhM Promotes Apoptosis of Macrophage and Facilitates Mtb Dissemination During Infection

**Figure 5:**
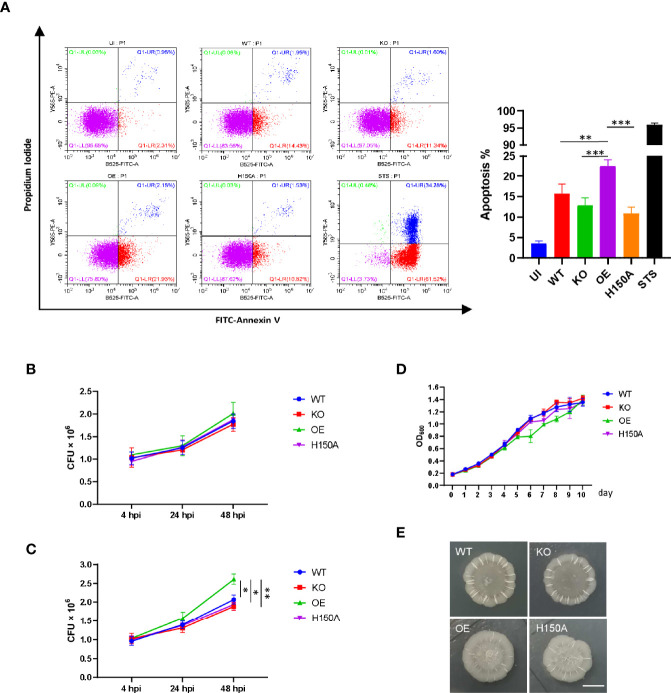
CdhM promotes apoptosis of macrophage and replication of Mtb during infection. **(A)** Apoptosis of RAW264.7 infected with CdhM-related strains was assessed using the FITC Annexin V Apoptosis Detection Kit (BD). Analysis was performed by flow cytometry (CytoFLEX LX, Beckman). Statistical analysis was showed on the right. STS (staurosporine) was used as positive control. **(B, C)** Colony-forming unit (CFU) of CdhM-related strains during infection of RAW264.7 were measured. Extracellular bacteria were washed at 4 h postinfection (hpi), then mediums with **(B)** or without **(C)** 50 µg/ml gentamycin were added for subsequent cell culture. The CFU of bacteria was measured at 24 and 48 hpi, respectively. **(D)** Growth curves of CdhM-related strains were measured by absorbance (OD600). **(E)** Colony morphology of CdhM-related strains on 7H10 agar plate. Scale bar, 5 mm. UI, uninfected. WT, wild-type H37Ra. KO, CdhM-knockout strain. OE, CdhM overexpression strain. H150A, CdhM-knockout strain complement with H150A mutant. *p < 0.05, **p < 0.01, ***p < 0.001 by two-way ANOVA. Means ± SD were shown (n = 3). Data are representative from at least three repetitions.

Since the ability of CdhM to induce ER stress during infection had been verified and the upregulation of CHOP in this process was observed too, we accordingly speculated that CdhM might also promote the apoptosis of macrophage during Mtb infection. Hence, the apoptosis of RAW264.7 infected with CdhM-related strains was assessed using FITC Annexin V Apoptosis Detection Kit (BD, USA), and analysis was performed by flow cytometry (CytoFLEX LX, Beckman). As shown in **Figure 5A**, infection with Mtb notably elevated the apoptosis of RAW264.7 compared to uninfected cells. The percent of apoptosis of CdhM-knockout and H150A mutant was less than that of the wild type but has no statistically significant differences, but they all were less than the CdhM-overexpression strain with statistically significant differences. It corresponds with the results before and proves that CdhM enhances apoptosis of macrophage during Mtb infection.

It is well defined that apoptosis of macrophages would influence the survival of infecting pathogens. Thus, we measured the colony-forming unit (CFU) of CdhM-related strains during infection of RAW264.7. Firstly, at 4 h postinfection (hpi), extracellular bacteria were washed with PBS and fresh mediums with 50 μg/ml gentamycin were added for continuing cell culture. The CFU of bacteria was measured at 24 and 48 hpi, respectively. As shown in **Figure 5B**, beyond our expectation, there are no obvious differences between all strains. It has already been discussed that apoptosis of macrophages is a double-edged sword during Mtb infection. In general, apoptosis restricts Mtb infection by releasing them into the extracellular environment in the form of apoptotic bodies. These bodies can be phagocytized by other macrophages for the further degradation of Mtb or by the dendritic cells, thereby leading to the activation of T lymphocytes. However, if the following actions are restrained, things might lead to another outcome. Thus, we tried to add mediums without antibiotics for subsequent cell culture after removing the extracellular bacteria at 4 hpi and measured the CFU at 24 and 48 hpi as before. As shown in **Figure 5C**, compared to wild-type or knockout strains, overexpression of CdhM conferred a proliferative advantage to Mtb during infection of macrophages *in vitro*. It should be noted that there were no obvious differences in growth curves and colony morphology among CdhM-related strains (**Figures 5D, E**). Thus, the proliferative advantage should be provided by CdhM-induced apoptosis that releases Mtb into the antibiotic-free extracellular environment. Taken together, CdhM may favor Mtb dissemination and proliferation at appropriate situations, like late stage of granulomas, by promoting ER stress-mediated apoptosis of infected macrophages, which liberates them into the extracellular milieu.

## Discussion

According to the Global Tuberculosis Report 2021, still about a quarter of the world’s population is infected with *Mycobacterium tuberculosis*. Until the COVID-19 pandemic, tuberculosis (TB) was the leading cause of death from a single infectious agent, ranking above AIDS. TB not only seriously endangers human health and life but also heavily increases the social burden, especially in poverty-stricken areas. It is therefore urgent to end TB. Unveiling the molecular mechanisms of the interaction of humans and Mtb is an indispensable theoretical basis for TB prevention and treatment. When Mtb invades the host, it is first recognized and internalized by macrophages, in which Mtb may interfere with the normal operation of multiple organelles, like the mitochondrion (Lee et al., 2021; Marchi et al., 2021), the lysosome (Wong et al., 2011), and the ER (Grover et al., 2018), *via* effector proteins, and result in loss of mitochondrial membrane potential, restriction of lysosomal acidification, and ER stress, respectively. When ER stress is induced, the UPR will be triggered to restore the ER homeostasis. Failure to restore the ER homeostasis leads to cell apoptosis.

In general, apoptosis of macrophages is considered as a tactic of the host to eliminate the infection pathogens. However, situations become more complicated when the macrophage is infected by Mtb (Aguilo N. et al., 2013). In some cases, Mtb can suppress apoptosis of the infected macrophages and remain latent in macrophages (Poirier et al., 2014), while in other cases, such as late stages of infection or in the caseous granulomas, Mtb can induce apoptosis of infected macrophages actively, thereby liberating them into the extracellular milieu and promoting their dissemination (Aguilo J. I. et al., 2013). As a result, apoptosis of macrophages is a double-edged sword during Mtb infection. The ER stress-mediated apoptosis during Mtb infection may belong to the latter, as it was first identified in the caseous granulomas (Seimon et al., 2010).

In this study, we identified a novel Mtb effector protein CdhM involved in host–Mtb interaction. CdhM could localize to the ER and interfere with the ER homeostasis, termed ER stress, through which the apoptosis of infected macrophages was induced to promote the dissemination of Mtb. This study reveals another such example in which the function of organelles and the fate of macrophages are manipulated by infected Mtb, which supplements new theoretical foundations for the understanding of TB pathogenesis and clinical therapeutics.

As far as we know, CdhM is the first effector protein of Mtb that was verified to exactly localize to the ER and cause an abnormal ER morphology. Mtb protein HBHA was found to induce ER stress (Choi et al., 2013), but its subcellular localization was not characterized; hence, the molecular mechanism of induction of ER stress may be different with CdhM. Rv0297 is another protein from Mtb that was reported to induce ER stress, and it was confirmed to localize to the ER by immunostaining (Grover et al., 2018). Using continuous live cell tracing, we observed that CdhM localized to the ER and caused cell apoptosis eventually, which is a visualized and convincing evidence for mediating apoptosis by CdhM-induced ER stress. It is worth mentioning that a single amino acid mutant of CdhM, including L44A, G96A, H150A, or W175A, is unable to induce ER stress and apoptosis, even though L44A, G96A, and W175A still localize to the ER, which implicates that these amino acids have a function necessary for CdhM induction of ER stress. Moreover, mutant H150A abolishes the ER localization, indicating that H150 may be involved in signal motifs for the ER localization of CdhM, but the precise signature motifs remain to be identified. CdhM is annotated as CDP-diacylglycerol pyrophosphatase in KEGG, which is an enzyme involved in lipid metabolism. As we all know, lipid synthesis is one of the functions of the ER. Whether induction of ER stress by CdhM is related to the annotated enzyme activity or some functions unknown remains to be further investigated.

Previous studies have reported that the expression of numerous ER stress-related genes changed in the TB granulomas (Seimon et al., 2010). In this study, we confirmed the results *in vitro* by transcriptome analysis of CdhM-transfected or -infected host cells. The results of our transcriptome analysis not only verified that Mtb induces ER stress during infection but also identified a single factor from Mtb, which means CdhM can make it. Subsequent biochemical experiments also validated it. In addition, the CFU assays of CdhM-related strain infection of macrophage proved that the apoptosis mediated by CdhM-induced ER stress promotes dissemination and proliferation of Mtb *in vitro*, which is a direct evidence for the physiological significance of induction of ER stress in TB granulomas. Although CdhM is not essential for Mtb growth in culture medium, similarly with mostly effector proteins in many pathogens, it may play an important role during infection. Future experiments in TB granulomas may provide a better insight into the role of CdhM.

Mtb commands effector proteins to interfere with the homeostasis of host cells for proliferation and dissemination, but it does not mean the host will go down without a fight. For example, BAG2 (BCL2-associated athanogene 2) was recently identified to ameliorate ER stress-mediated apoptosis of macrophage during Mtb infection through selective autophagy (Liang et al., 2020). Therefore, the arms race between Mtb and humans is continuing, and more underlying information about the host–Mtb interaction remains to be explored for clinical TB therapeutics.

## Data Availability Statement

The data presented in the study are deposited in the NCBI repository, accession number are PRJNA807506 (HEK293T) and PRJNA808069 (RAW264.7).

## Author Contributions

Z-GH and PX conceived the study. PX designed and performed all experiments, assisted by JT. PX analyzed the data and wrote the manuscript. Z-GH reviewed the manuscript and supervised the research. All authors contributed to the article and approved the submitted version.

## Funding

This work was supported by the National Natural Science Foundation of China (31730005), National Key R&D Program of China (2020YFA0907200), and Ba-Gui Scholar Program of Guangxi (To Z-GH).

## Conflict of Interest

The authors declare that the research was conducted in the absence of any commercial or financial relationships that could be construed as a potential conflict of interest.

## Publisher’s Note

All claims expressed in this article are solely those of the authors and do not necessarily represent those of their affiliated organizations, or those of the publisher, the editors and the reviewers. Any product that may be evaluated in this article, or claim that may be made by its manufacturer, is not guaranteed or endorsed by the publisher.
