# Supplementary material for: Induction of Endoplasmic Reticulum Stress by CdhM Mediates Apoptosis of Macrophage During Mycobacterium tuberculosis Infection
Source: Front Cell Infect Microbiol. 2022 Apr 4;12:877265. doi: 10.3389/fcimb.2022.877265 (PMC9013901; doi:10.3389/fcimb.2022.877265)
Supplement: Supplementary file 1 [file DataSheet_1.pdf]

Supplementary Material

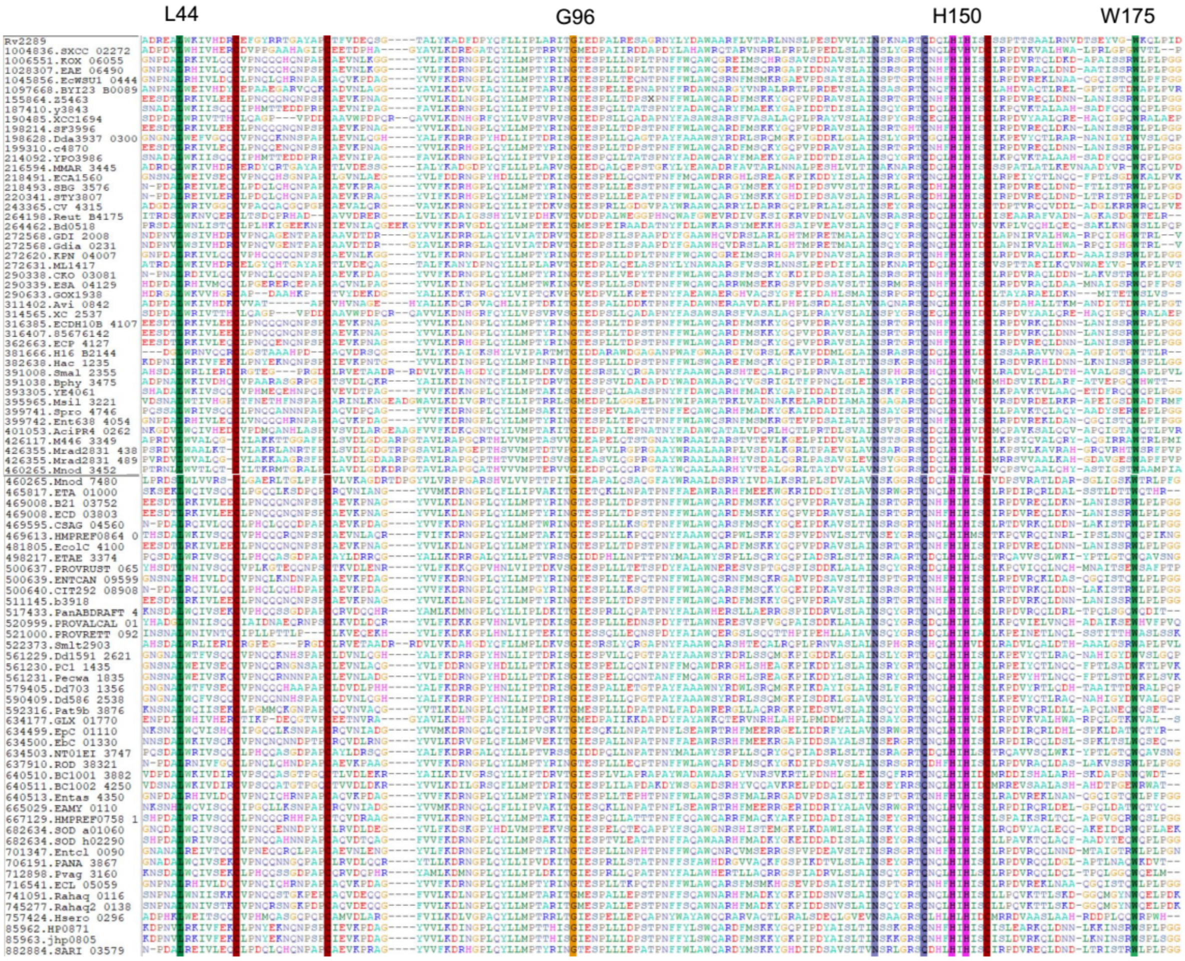

Supplementary Figure 1. Conserved amino acids of CdhM (Rv2289) among all homologous sequences. Analyzed by ClustalW Multiple Alignment in BioEdit.

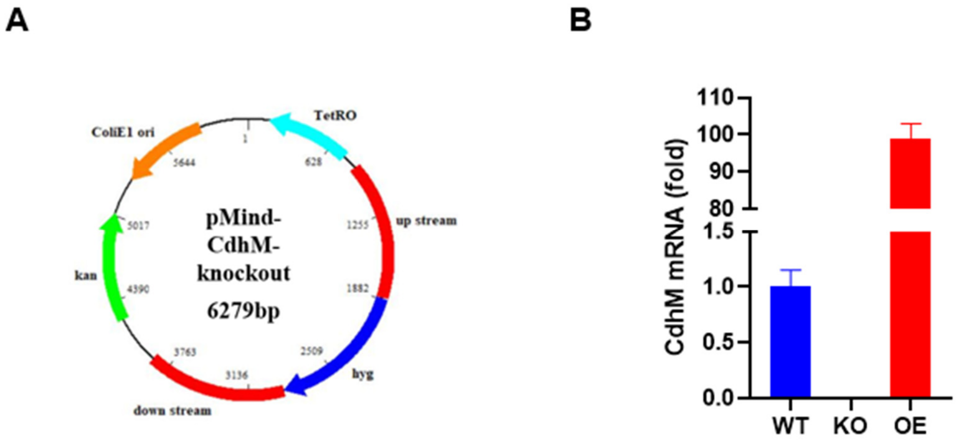

Supplementary Figure 2. **(A)** Plasmid map of pMind-CdhM-knockout, which was used for CdhM knockout from the genome of H37Ra. **(B)** Transcript levels of CdhM in CdhM-related strains. Measured by qRT-PCR. SigmaA was used as internal reference.

Supplementary Movies S1-4. Time-lapse live imaging to trace the behavior of the cells transfected with GFP-CdhM. **S1**, empty vector pEGFP-C1. **S2**, pEGFP-C1-CdhM. **S3**, pEGFP-C1-G96A. **S4**, pEGFP-C1-H150A.

Table S1 Statistics for the RNA-Seq data of RAW264.7. Each group with three parallel replicates. M, million. Q30 %, the percentage of nucleotides with a quality value of 30. GC %, the percentage of guanine and cytosine in the cleaned reads.

Sample	Raw_Reads	Clean_Reads	Clean_Bases	Q30_%	GC_%	Total_Map	Unique_Map	Multi_Map
UI-1	42.81M	41.48M	6.22G	93.33	51.35	91.17%	85.51%	5.67%
UI-2	42.75M	41.70M	6.25G	92.95	51.62	91.07%	85.49%	5.58%
UI-3	52.31M	51.14M	7.67G	93.21	51.36	91.51%	85.62%	5.90%
WT-1	45.59M	44.66M	6.7G	92.78	51.19	91.22%	85.17%	6.04%
WT-2	45.64M	44.88M	6.73G	92.47	51.08	90.87%	84.96%	5.90%
WT-3	55.97M	55.05M	8.26G	92.93	50.91	91.36%	85.23%	6.14%
KO-1	54.09M	53.20M	7.98G	90.43	50.86	89.98%	84.08%	5.90%
KO-2	42.08M	41.35M	6.2G	92.96	50.56	91.41%	85.26%	6.15%
KO-3	45.94M	45.16M	6.77G	92.51	51.27	91.01%	85.15%	5.86%
OE-1	47.82M	46.99M	7.05G	92.25	51.35	90.90%	85.05%	5.86%
OE-2	57.02M	55.98M	8.4G	92.53	51.48	90.95%	85.03%	5.92%
OE-3	44.78M	44.07M	6.61G	92.68	51.22	91.15%	85.30%	5.85%

Table S2 Primers for qRT-PCR in this study.

BiP-F	GAAAGAAGGTTACCCATGCAGT
BiP-R	CAGGCCATAAGCAATAGCAGC
hCHOP-F	GGAAACAGAGTGGTCATTCCC
hCHOP-R	CTGCTTGAGCCGTTCATTCTC
mCHOP-F	CTCGCTCTCCAGATTCCAGTC
mCHOP-R	CTTCATGCGTTGCTTCCCA
hGAPDH-F	GAGTCAACGGATTTGGTCGT
hGAPDH-R	GACAAGCTTCCCGTTCTCAG
mGAPDH-F	CCTTGACTGTGCCGTTGAATTT
mGAPDH-R	CACCTATGGTGCAACAGTATTCC
hXBP1-F	TTACGAGAGAAAACTCATGGCC
hXBP1-R	GGGTCCAAGTTGTCCAGAATGC
mXBP1-F	ACACGCTTGGGAATGGACAC
mXBP1-R	CCATGGGAAGATGTTCTGGG
CdhM-F	tgttccgggacatctacctg
CdhM-R	cagtcgtggtcttggagagt
SigmaA-F	TCGCGCCTACCTCAAACAG
SigmaA-R	GCTACAGGCCAGCCTCGAT
